# Construction and characterization of an expressed sequenced tag library for the mosquito vector *Armigeres subalbatus*

**DOI:** 10.1186/1471-2164-8-462

**Published:** 2007-12-18

**Authors:** George F Mayhew, Lyric C Bartholomay, Hang-Yen Kou, Thomas A Rocheleau, Jeremy F Fuchs, Matthew T Aliota, I-Yu Tsao, Chiung-Yen Huang, Tze-Tze Liu, Kwang-Jen Hsiao, Shih-Feng Tsai, Ueng-Cheng Yang, Nicole T Perna, Wen-Long Cho, Bruce M Christensen, Cheng-Chen Chen

**Affiliations:** 1 Department of Pathobiological Sciences, University of Wisconsin-Madison, 1656 Linden Dr., Madison Wisconsin, 53706, USA; 2 Department of Entomology, Iowa State University, Ames IA, 50010, USA; 3 Department of Tropical Medicine, National Yang-Ming University, Shih-Pai, Taipei 112, Taiwan; 4 VYM Genome Research Center, National Yang-Ming University, Shih-Pai, Taipei 112, Taiwan; 5 Department of Medical Research and Education, Taipei Veterans General Hospital, Taipei 112, Taiwan; 6 Division of Molecular and Genomic Medicine, National Health Research Institutes, Miaoli County 350, Taiwan; 7 Institute of Bioinformatics, National Yang-Ming University, Shih-Pai, Taipei 112, Taiwan

## Abstract

**Background:**

The mosquito, *Armigeres subalbatus*, mounts a distinctively robust innate immune response when infected with the nematode *Brugia malayi*, a causative agent of lymphatic filariasis. In order to mine the transcriptome for new insight into the cascade of events that takes place in response to infection in this mosquito, 6 cDNA libraries were generated from tissues of adult female mosquitoes subjected to immune-response activation treatments that lead to well-characterized responses, and from aging, naïve mosquitoes. Expressed sequence tags (ESTs) from each library were produced, annotated, and subjected to comparative analyses.

**Results:**

Six libraries were constructed and used to generate 44,940 expressed sequence tags, of which 38,079 passed quality filters to be included in the annotation project and subsequent analyses. All of these sequences were collapsed into clusters resulting in 8,020 unique sequence clusters or singletons. EST clusters were annotated and curated manually within ASAP (A Systematic Annotation Package for Community Analysis of Genomes) web portal according to BLAST results from comparisons to Genbank, and the *Anopheles gambiae *and *Drosophila melanogaster *genome projects.

**Conclusion:**

The resulting dataset is the first of its kind for this mosquito vector and provides a basis for future studies of mosquito vectors regarding the cascade of events that occurs in response to infection, and thereby providing insight into vector competence and innate immunity.

## Background

The perpetuation of mosquito-borne diseases is dependent on the compatibility of the pathogen with its invertebrate and vertebrate hosts, as dictated by each respective genome. The failure of traditional mosquito-borne disease control efforts to reduce the burden of these diseases on public health has created an incentive to develop a more comprehensive understanding of molecular interactions between host and pathogen, in order to develop novel means to control disease transmission. Innate immune responsiveness in the mosquito host is of particular interest in such explorations because extensive research efforts have shown that vector mosquito species produce robust humoral and cellular immune responses against invading pathogens [1-4].

A vector species that employs a unique, robust immune response against an invading pathogen is the mosquito, *Armigeres subalbatus*, a natural vector of the nematode parasites that cause lymphatic filariasis. This debilitating disease affects 120 million people annually, one third of who suffer gross pathology (CDC 2006). *Ar. subalbatus *is ideally suited for laboratory studies of immune responsiveness because it is a natural vector of the filarial worm, *Brugia pahangi*, but it exhibits a refractory state to the microfilariae of *Brugia malayi *by virtue of a strong melanotic encapsulation response; therefore, it is the ideal organism for studying molecular mechanisms of the anti-filarial worm response as a function of the broader innate immune capacity of the mosquito. In fact, *Ar. subalbatus *is one of the few species of mosquito to effectively use melanotic encapsulation as a natural defense mechanism against metazoan pathogens [5]. *Ar. subalbatus *also serves as a competent laboratory vector of *Plasmodium gallinaceum*, the causative agent of avian malaria in Asia ([6] and Christensen *et al.*, unpublished data), and also has been implicated in the transmission of Japanese encephalitis virus in Taiwan [7,8].

Experimental evidence has shown that humoral and cellular immune responses play a fundamental role in mosquito refractoriness to a particular pathogen; however, very little is known about their genetic control. As a result, our laboratory is using Expressed Sequence Tags (ESTs) as a tool to elucidate the function of known genes and assist in the discovery of previously unknown, "immunity"-related genes. In addition, this high-throughput molecular approach to gene discovery provides the capacity to tactically design oligonucleotide-based microarrays that can be further used to gain insight into vector-pathogen interactions. With no genome sequencing project on the horizon for *Ar. subalbatus*, these EST libraries and microarrays constitute the only tools currently available to gauge immune responsiveness in this medically important vector species.

We previously reported a comprehensive analysis of ESTs from complementary DNA (cDNA) libraries created from adult, female *Ar. subalbatus *hemocytes [1]. Experimental evidence has shown the importance of mosquito hemocytes (blood cells) as both initiators and mediators of mosquito immune responses [9-13]; therefore, material was collected from the perfusate (which contains hemocytes) of *Micrococcus luteus *and *Escherichia coli *inoculated mosquitoes at 1, 3, 6, 12, & 24 hours post bacterial inoculation. These bacterial species have been extensively used to examine immune peptide production in mosquitoes [14,15], and each activates a different arm of the innate immune response. The primary response of *Ar. subalbatus *to *E. coli *is phagocytosis, whereas the primary response to *M. luteus *is melanization, and it has been determined that this is independent of Gram type [10,11].

In order to more completely represent the baseline physiology and innate immune capabilities of this mosquito, cDNA libraries were created from adult, female *Ar. subalbatus *mRNA collected from whole body mosquitoes inoculated with the same mixture of bacteria. Material also was collected from whole body *Ar. subalbatus *exposed to filarial worm parasites. A blood meal containing *B. malayi *induces the melanization response in *Ar. subalbatus*; therefore, whole body material was collected from female mosquitoes 24, 48, and 72 hours after an infective blood feed. Intrathoracic injection of *Dirofilaria immitis *microfilariae into the mosquito's hemocoel also induces a strong melanotic encapsulation response in *Ar. subalbatus *and is a model system by which the immune response is stimulated without exposing the mosquito to both the parasite and a blood meal [16]. This model system for infection facilitates the uncoupling of two processes – namely blood meal digestion and ovarian development – that compete for biochemical resources [17]. Whole body mosquitoes inoculated with *D. immitis *were collected at 24 and 48 hours post-inoculation. Libraries also were constructed from 5–7 and 14–21 day old naïve whole body females to ensure representation of transcripts from non-immune activated, aging mosquitoes. An attempt to sequence clones from a library from blood-fed naïve females was not successful.

## Results and Discussion

### Sequencing and clustering

Non-normalized cDNA libraries were constructed from newly emerged female mosquitoes inoculated with bacteria, inoculated or blood fed with filarial worm parasites, and from aging, naïve adult females. ESTs were sequenced from the 5' end by the University of Wisconsin Genome Sequencing Center, and the National Yang-Ming University core facility, and were assembled to collapse the entire dataset, reduce redundancy, and simplify downstream annotation (Table 1). Of the 44,940 trace files generated by the two sequencing units, 38,079 traces passed quality control (85% success rate) and were sent to assembly with an average high quality (phred score 20+) length of 450 bases. The resulting collapsed data resulted in 8,020 clusters, of which 4,949 are composed of one trace (singletons). The deepest cluster contains 870 ESTs, with an average of 11 (+/- 37) ESTs per cluster.

**Table 1:** Summary of *Ar. subalbatus* EST and EST cluster production from six cDNA libraries.

	# ESTs	# EST clusters
Bacteria (whole body)	5,654	2,372
Bacteria (hemocytes)	11,300	2,107
*D. immitis *(whole body)	7,014	2,510
*B. malayi *(whole body)	7,192	3,096
Naïve 7 (whole body)	4,289	1,911
Naïve 14 (whole body)	5,405	2,325
Combined	38,079	8,020

The condition of mosquitoes/inocula used to generate the material for each cDNA library is listed in the left-hand column. Bacteria = mixture of *E. coli *and *M. luteus*.

### Functional annotation of EST clusters and singletons

Consensus sequences from clustering were output in fasta format and used in comparisons to the GenBank non-redundant database, the *D. melanogaster *and *An. gambiae *genomes, and to the other *Ar. subalbatus *EST sets created during the project.

Each EST cluster/singleton and its corresponding sequence similarity data were uploaded into ASAP. Within the ASAP interface, annotators assessed sequence alignments and followed intact hyperlinks to NCBI, the Wellcome Trust Sanger Institute (Ensembl), FlyBase, and orthologous sequences within ASAP and at National Yang Ming University, in order to ascribe a predicted gene product and/or function to each sequence. Supporting evidence for each annotation is typically in the form of a hyperlink to a database and can be viewed in ASAP. These annotations then were reviewed and approved or rejected by a curator. Annotation followed the controlled vocabulary established in a previous study, such that each EST cluster was attributed with some functional information, and indication of quality of the BLAST hit used to attribute that information [1]. Of the 8,020 EST clusters, 2,843 were annotated as "unknown" (having no significant match to any of the databases searched), and 1896 were annotated as "conserved unknown" with varying degrees of confidence. Sequences were submitted to NCBI as annotated EST clusters into the Core Nucleotide database and made available for public viewing through ASAP.

### Library to library comparison

**Figure 1:**
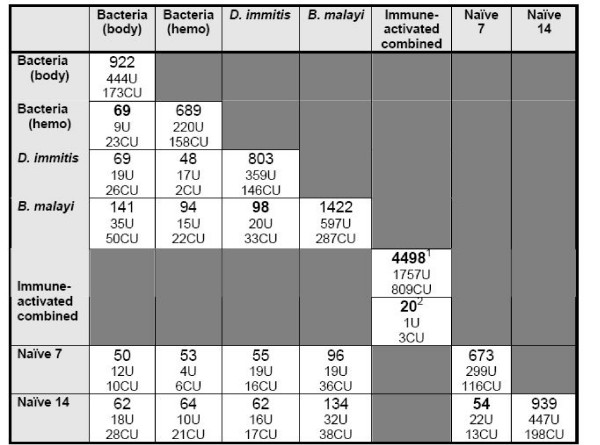
**A comparison of EST clusters from *6 Ar. subalbatus *cDNA libraries (8,020 clusters total)**. The type of immune response activation for mosquitoes is listed in the primary row and column. At the intersection of each row and column, the number of clusters unique to that combination of libraries is listed in bold, followed by the number of those clusters that are designated as unknown (U) or conserved unknown (CU). Clusters from the 4 immune response activated libraries (Immune activated combined) were queried against the naïve libraries such that: a cluster is represented in at least 1 of the 4 libraries (but not in naive) (top -1), or clusters are represented in all 4 of the libraries (but not in naive) (bottom – 2).

An analysis of EST clusters from the complete project was done by combining annotations and cluster composition (in terms of source libraries) to provide insight into the molecular effort put forth by the mosquito in the face of different types of immunological challenge. Within Microsoft Access, a table was built that contains EST clusters according to ASAP ID number, contig number (created during assembly in Seqman), project (cDNA library) from which ESTs were contributed, and the number of ESTs contributed per project. Queries were built to extract the number of EST clusters unique to a particular library (e.g., bacteria-inoculated whole body), or shared between projects (e.g. bacteria-inoculated whole body and hemocyte libraries) (Figure 1). Shared are 69 clusters unique to a response to bacteria-inoculation, 98 unique to the response against filarial nematodes, and 4,498 are represented in at least one of the 4 immune-activated projects. Amongst those 4,498, 20 are represented in all 4 of those projects, perhaps indicative of the importance of these genes in immune responsiveness. Included amongst these 20 is a Clip domain serine protease (*An. gambiae *[ENSANGP00000017225]), Serpin 27A (D. melanogaster [FBgn0028990]), and Aslectin (AY426975) – a ficolin-like pattern recognition molecule [18]. Unique to the response against *B. malayi *infection is a protein-tyrosine kinase, involved in the JAK-STAT cascade, which is represented by 107 ESTs.

To examine the statistical likelihood that the numbers of ESTs in each cluster represent a true sampling of the biological variation between the six libraries, and to compare the results of clustering with microarray results [19], cluster data were submitted to the IDEG6 website for analysis [20]. The number of ESTs in each cluster was normalized based on the number of total ESTs collected and the total number of ESTs in each library. Six statistics were compared, including Audic and Claverie [21], Greller and Tobin [22], Stekel [23], Chi Square 2 × 2, general Chi Square, and Fisher's Exact Test, all corrected via a Bonferroni method. The results of the entire test are included as a supplementary table (see Additional file 1). Table 2 presents the 99 EST clusters that show the highest significant difference between libraries (p > 0.00001, R > 4). Although significant increases in ESTs encoding immune-related products are observed (i.e. sequences expected to be increasing according to infection status of the mosquitoes used to collect material for libraries), this is not always the case. Several clusters that encode "house keeping" products are demonstrably enriched for ESTs from immune-challenged libraries (e.g. cytochromes and dehydrogenases) suggesting that these metabolic genes play essential roles in the physiology of an immune and/or stress response. In addition, many of the clusters that are significantly different between libraries encode gene products of completely unknown function. A comparison with microarray data from Aliota, *et al.*[19], shows some overlap between the two methods. Out of the 99 clusters that are significantly different (Table 2), 19 share significant changes when compared with microarray data from *B. malayi *infected females (highlighted in Table 2). Combined, these EST and microarray data provide several target sequences for further study in relation to mosquito innate immunity.

**Table 2:** Clusters showing significant differences as determined by Stekel R value and Chi square analysis.

**GBID**	**Product**	**asuhem**	**diroinf**	**imacbac**	**brumal**	**naïve7**	**naïve14**	**R**	**Chi**
EU204979	NADH dehydrogenase subunit 2	245	23	14	10	21	19	0	0
EU204980	NADH dehydrogenase subunit 6	172	18	8	1	10	11	0	0
EU204999	cytochrome c oxidase subunit I	245	67	24	11	59	54	0	0
EU205005	unknown	325	52	33	17	26	66	0	0
EU205016	cytosolic small ribosomal subunit S26	60	69	57	41	36	67	0.000001	0.000001
EU205017	NADH dehydrogenase	28	2	2	4	6	1	0.000002	0.000001
EU205057	ubiquitin	83	0	0	0	1	0	0	0
EU205058	cytochrome c oxidase subunit III	277	79	83	20	62	52	0	0
EU205059	cytochrome b	47	19	14	1	9	8	0	0
EU205061	mitochondrial adenosine triphosphatase subunit 6	202	60	36	1	51	45	0	0
EU205096	ubiquinol-cytochrome c reductase subunit	62	9	4	12	9	11	0	0
EU205097	cytochrome c	74	15	8	12	11	12	0	0
EU205100	unknown	243	59	123	79	54	72	0	0
EU205101	putative: conserved unknown	32	18	7	3	20	6	0.000001	0.000002
EU205152	ubiquinol-cytochrome c reductase	56	14	5	11	14	9	0	0
EU205153	AT DNA binding	0	7	15	13	6	8	0.000002	0.00033
EU205154	questionable: Bombyx mori prophenoloxidase activating factor 3	26	1	3	3	1	1	0.000001	0
EU205155	questionable: conserved unknown	1	6	7	21	8	10	0.000006	0.00008
EU205171	ubiquinol-cytochrome-c reductase	94	16	8	29	20	26	0	0
EU205182	cytosolic large ribosomal subunit L36	27	43	33	24	26	47	0	0
EU205185	NADH dehydrogenase	47	2	8	10	4	7	0	0
EU205197	gelsolin	129	20	38	33	13	57	0	0
EU205226	cytosolic large ribosomal subunit L40	21	5	0	0	5	1	0	0.000005
EU205248	cytochrome C oxidase subunit II	52	15	12	6	9	27	0.000001	0.000002
EU205296	Myosin alkali light chain 1	35	3	8	0	3	6	0	0
EU205304	questionable: receptor binding	0	0	0	2	6	0	0.000228	0.000002
EU205306	questionable: cytochrome c oxidase	63	13	6	6	13	3	0	0
EU205324	conserved unknown	17	0	4	0	0	4	0.000003	0.000054
EU205330	cytosolic small ribosomal subunit S9	39	73	43	68	32	54	0	0
EU205338	superoxide dismutase	20	2	1	0	1	1	0.000002	0.000001
EU205349	ribosomal protein L41	276	100	170	94	126	104	0	0
EU205374	nucleoside diphosphate kinase	2	14	1	11	0	5	0.000002	0.000002
EU205385	questionable: protein-tyrosine kinase	0	0	0	107	0	0	0	0
EU205393	hydrogen transporting two sector ATPase	53	11	11	8	17	9	0.000003	0.000002
EU205409	cecropin	17	1	1	0	0	5	0.000006	0.000023
EU205450	serine protease	1	6	28	9	3	19	0	0
EU205451	cytosolic small ribosomal subunit S3A	78	26	27	16	20	29	0.000068	0.000132
EU205465	defensin	136	14	27	6	5	9	0	0
EU205470	putative: heat shock	0	0	1	56	2	3	0	0
EU205505	lysozyme	116	17	32	10	11	35	0	0
EU205526	cytochrome c oxidase	130	15	11	10	16	5	0	0
EU205556	putative: conserved unknown	0	0	3	41	0	3	0	0
EU205557	putative: conserved unknown	0	0	0	9	0	1	0.000047	0.000003
EU205560	cecropin	32	35	17	3	14	11	0	0
EU205570	putative: conserved unknown	0	9	13	15	7	7	0.000003	0.000718
EU205592	conserved unknown	63	21	11	12	12	16	0.000007	0.000004
EU205632	putative: calcium ion binding protein	0	0	13	0	0	0	0	0
EU205658	serine protease	112	18	42	43	7	29	0	0
EU205659	unknown	35	0	3	0	2	0	0	0
EU205708	proton-transporting ATP synthase complex subunit	70	8	9	4	14	16	0	0
EU205709	putative: conserved unknown	25	0	3	2	2	2	0.000001	0
EU205750	hydrogen-exporting ATPase	33	2	5	2	1	9	0	0
EU205751	putative: odorant-binding	0	0	0	9	0	0	0.000015	0
EU205764	questionable: odorant-binding protein 56e	0	0	0	9	0	0	0.000015	0
EU205767	trypsin	0	0	0	25	0	0	0	0
EU205782	trypsin	10	221	98	70	107	70	0	0
EU205800	unknown	5	4	9	7	11	39	0	0
EU205806	questionable: odorant-binding	0	0	0	123	0	0	0	0
EU205807	questionable: odorant-binding	0	0	0	28	0	0	0	0
EU205828	trypsin	3	85	10	11	33	2	0	0
EU205832	questionable: apolipophorin	129	0	5	32	2	6	0	0
EU205858	vitellogenin C	0	0	0	18	0	0	0	0
EU205893	unknown	130	0	0	0	0	1	0	0
EU205915	hydrogen transporting two sector ATPase	41	6	5	2	2	4	0	0
EU205924	putative: serine protease	5	13	28	2	14	6	0	0
EU205925	putative: serine protease	1	15	10	4	12	5	0.000001	0.000002
EU205991	unknown	0	3	1	39	2	4	0	0
EU205996	ATPase synthase	16	4	1	0	0	0	0.000003	0.000012
EU206001	NADH dehydrogenase	36	6	3	8	7	1	0.000001	0.000001
EU206048	conserved unknown	45	8	11	10	4	6	0.000021	0.000005
EU206083	putative: serine protease	0	0	0	61	0	0	0	0
EU206123	putative: serine protease	0	5	11	28	7	8	0	0
EU206138	questionable: conserved unknown	0	0	0	32	0	0	0	0
EU206244	cytosolic large ribosomal subunit L34a	1	38	10	9	7	3	0	0
EU206248	zinc-metalloproteinase precursor	2	14	1	0	12	3	0	0
EU206376	putative: cathepsin	0	0	0	30	0	0	0	0
EU206422	infection responsive short peptide (gambicin)	17	23	40	47	17	24	0.000001	0.000002
EU206504	unknown	0	0	0	12	0	0	0	0
EU206507	ATP/ADP antiporter (transporter)	21	3	1	0	1	0	0	0
EU206546	beta-globin	0	0	0	34	0	0	0	0
EU206601	cathepsin	0	0	0	19	0	0	0	0
EU206635	trypsin	0	4	0	16	3	2	0	0
EU206733	putative: conserved unknown	0	0	0	9	0	0	0.000015	0
EU206824	putative: arrestin 2	0	13	6	8	9	9	0.000004	0.000307
EU206866	questionable: conserved unknown	0	0	0	32	0	0	0	0
EU206900	rhodopsin	0	2	13	11	5	11	0.000001	0.000066
EU206907	questionable: chitin binding	0	0	0	12	0	0	0	0
EU206986	unprocessed 18S ribosomal RNA	40	1	0	1	0	0	0	0
EU207626	unknown	90	0	0	1	0	2	0	0
EU207634	unknown	107	56	65	38	22	89	0	0
EU207658	NADH dehydrogenase subunit	26	2	2	1	8	5	0.000003	0.000006
EU207722	questionable: triacylglycerol lipase	0	0	0	8	0	0	0.000066	0.000002
EU207765	putative: odorant-binding protein G.1A.F	0	0	0	22	0	0	0	0
EU207810	putative: trypsin	0	0	0	21	1	0	0	0
EU207816	hydrogen transporting two sector ATPase	48	8	8	6	8	13	0.000004	0.000001
EU207921	vitellogenin	0	0	0	19	0	0	0	0
EU207965	defensin	17	2	5	0	0	0	0.000002	0.00002
EU208023	questionable: threonine-rich salivary mucin	0	0	0	0	6	4	0.000031	0.000003
EU212979	NADH dehydrogenase	36	5	10	0	4	3	0	0

Clusters and their constituent ESTs were analysed using IDEG6 [20] to find clusters where the number of ESTs were statistically different between the libraries. Of 8,020 clusters analysed, 99 showed a significantly differential number of ESTs collected from one or more of the libraries. Each row represents the Genbank Accession number for cluster, and the columns are the number of ESTs from each of the six libraries that are a member of it. The libraries are: asuhem (bacteria-inoculated, hemocyte), diroinf (whole body, *Dirofilaria immitis *injected), imacbac (bacteria-inoculated whole body), brumal (blood-fed *Brugia malayi*), n7 and n14 (naïve, 5–7 and 12–14 days of age). The R value is the inverse log of the Stekel R Score [23], and the Chi value is a general Chi square analysis.

### Gene ontology

**Figure 2:**
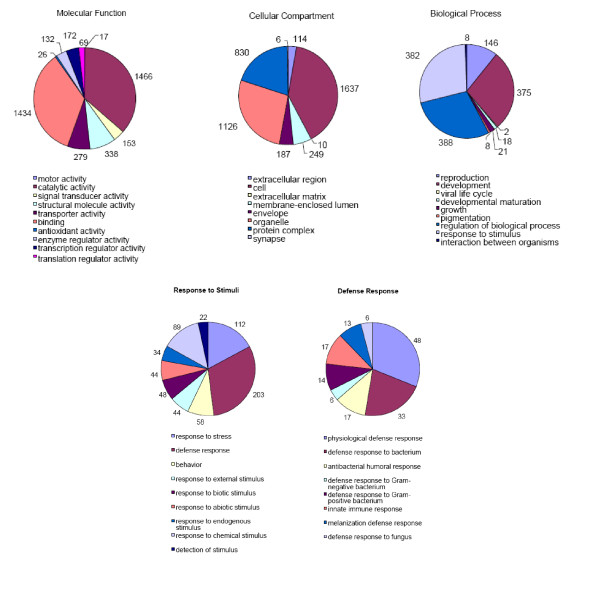
**Summary of Gene Ontology assignments to 2,793 *Ar. subalbatus *clusters**. A gene list of assigned annotations was processed using WEGO (Web Gene Ontology Annotation Plot). Numbers outside the pie charts represent the number of clusters within each category. (Top) Tier 2 summaries of the three main branches of the gene ontology: Molecular Function, Cellular Component, and Biological Process. The categories of physiological process (GO:0007582) and cellular process (GO:0009987) were removed from the display in order to visualize the categories containing fewer members. (Bottom) Pie charts of Tier 3 and Tier 4, 5, and 6 of the GO subcategory of Biological Processes, response to stimuli (GO:0050896) and defense response.

To attribute more functional information to annotations in ASAP, Gene Ontology (GO) classifications were migrated from Flybase annotations to homologous *Ar. subalbatus *clusters, because Flybase contains the most complete dataset for a related species from which to draw. GO annotations were attributed to 2,793 (35% of total) EST clusters. From the perspective of the entire dataset, 851 (11%) clusters have annotations but lack a GO annotation. Data from GO analyses are presented graphically, according to second tier categories within the top-level categories of Biological Process, Cellular Compartment, and Molecular Function. Of particular interest for this dataset are those clusters related to innate immunity, so a more in-depth (4^th ^and 6^th ^tier) view is presented (Figure 2).

Because of the unique immune response capabilities of *Ar. subalbatus*, EST clusters were interrogated beyond the GO analysis for clusters encoding immunity-related proteins. Those clusters encoding proteins that have a documented role in *Ar. subalbatus *immunity were sorted according to representation in different libraries (Table 3). In addition, immunity related genes and proteins were subdivided into categories including: CASPs: Caspases, CATs: Catalases, CLIPs: CLIP-Domain Serine Proteases, CTLs: C-Type Lectins, FREPs: Fibrinogen-Related Proteins, GALEs: Galactoside-Binding Lectins, IAPs: Inhibitors of Apoptosis, IMDPATHs: IMD Pathway Members, JAKSTATs: Signal Transduction, LYSs: Lysozymes, MLs: MD2-Like Receptors, PGRPs: Peptidoglycan Recognition Proteins, PPOs: Prophenoloxidases, PRDXs: Peroxidases, REL: Relish-like Proteins, SCRs: Scavenger Receptors, SODs: Superoxide Dismutatses, SPZs: Spaetzle-like Proteins, SRPNs: Serine Protease Inhibitors, TEPs: Thio-Ester Containing Proteins, TOLLs: Toll-Receptors, and TOLLPATHs: Toll Pathway Members. Representatives of each of these subcategories can be found amongst the ESTs in these libraries (Tables 3 and 4). Clusters identified as immunity-related according to homology to genes in ImmunoDB [24] were broken down into the number of ESTs represented per cluster from each library (Table 4).

**Table 3:** Armigeres subalbatus EST clusters that represent characterized, published sequences from this mosquito.

**GB Accession**	**Description**	**ASAPID**	**asuhem**	**diroinf**	**imacbac**	**brumal**	**n7**	**n14**
AF318200.1	ABC membrane transporter (white)	n/a						
AY426976.1 (AY426975.1)	aslectin AL-1	ACN-0180799	3	1	1	1	0	0
		ACN-0183776	1	0	0	0	0	0
AY603183.1	beta 1,3-glucan recognition protein (GRP)	ACN-0184624	0	0	1	0	0	0
AY662686.1	dopa decarboxylase	ACN-0182608	0	2	1	0	1	0
AY960762.1	dopachrome conversion enzyme	ACN-0181349	0	1	0	0	0	1
AF317822.1	glucose-6-phosphate dehydrogenase (G6pd)	n/a						
AY571966.1	phenylalanine hydroxylase	ACN-0184263	1	0	0	1	1	1
AF260567.1	prophenoloxidase I	n/a						
AF286468.1	prophenoloxidase II	n/a						
AY487171.1	prophenoloxidase III	n/a						
AY487172.1	prophenoloxidase IV	ACN-0186737	0	0	0	1	0	0
DQ862064.1	prophenoloxidase V	ACN-0182951	0	0	1	0	0	0
DQ862065.1	prophenoloxidase VI	ACN-0186767	0	1	0	0	0	0
		ACN-0182961	1	0	0	0	0	0
AY487170.1	serine protease	ACN-0181859	0	0	0	1	0	0

Because of the unique, robust melanization response that is elicited when *Ar. subalbatus *is infected with certain nematode parasites, several molecules related to melanogenesis have been the subject of comprehensive characterization. EST clusters encoding these gene products are presented according to the number of ESTs in the following libraries: asuhem (bacteria-inoculated, hemocyte), diroinf (whole body, *Dirofilaria immitis *injected), imacbac (bacteria-inoculated whole body), brumal (blood-fed *Brugia malayi*), n7 and n14 (naïve, 7 and 14 days of age).

**Table 4:** Searching *Ar. subalbatus* EST clusters for immunity-related sequences based on homology to other flies.

**Family**	**Subfamily**	**asuhem**	**diroinf**	**imacbac**	**brumal**	**naïve7**	**naïve14**	**Total Reads**	**R Value**
AMP	Cecropin	98	65	70	56	41	47	377	0.070988
**AMP**	***Defensin***	***238***	***41***	***78***	***16***	***15***	***28***	***416***	***0***
AMP	Diptericin	0	2	0	0	0	1	3	0.160764
CASP	CASP	1	1	0	1	1	0	4	0.673041
CAT	CAT	0	1	0	0	0	0	1	0.561495
CLIP	CLIP-CLIP-CLIP-SPH	1	1	1	0	0	1	4	0.670142
**CLIP**	**CLIP-SP**	**136**	**33**	**37**	**24**	**22**	**27**	**279**	**0**
CLIP	CLIP-SP-CLIP-SP	1	0	0	1	0	0	2	0.668311
**CLIP**	**CLIP-SPH**	**153**	**33**	**55**	**58**	**14**	**47**	**360**	**0**
CLIP	SP-CLIP	4	0	0	0	0	0	4	0.060745
CLIP	SP-CLIP-SPH	2	0	0	1	0	0	3	0.440816
CLIP	SPH-CLIP	1	0	0	0	0	0	1	0.754901
CLIP	SPH-CLIP-SPH	1	1	0	2	0	1	5	0.576541
CLIP	SP-SPH-CLIP	0	1	0	0	0	0	1	0.561495
**CTL**	**CTL**	**80**	**19**	**27**	**17**	**25**	**17**	**185**	**0.000002**
**FREP**	**FREP**	**17**	**9**	**22**	**4**	**4**	**8**	**64**	**0.000764**
GALE	GALE	0	0	2	1	1	2	6	0.206431
GNBP	GNBP	0	1	1	0	0	0	2	0.420516
IAP	IAP	1	1	0	3	0	2	7	0.260929
IMDPATH	IKKg	1	0	0	1	0	0	2	0.668311
**LYS**	**LYSC**	**122**	**21**	**37**	**19**	**15**	**42**	**256**	**0**
ML	ML	14	10	5	8	9	3	49	0.223609
PGRP	PGRP	9	1	4	2	2	5	23	0.312906
PPO	PPO	1	1	0	1	0	0	3	0.653363
PRDX	GPX	8	2	1	1	0	3	15	0.100553
PRDX	HPX	1	2	1	3	1	0	8	0.446023
PRDX	TPX	7	3	5	14	4	8	41	0.136651
SCR	SCRA	0	7	3	1	2	2	15	0.004735
SCR	SCRB	1	0	0	1	2	0	4	0.26927
SCR	SCRC	1	0	0	0	0	0	1	0.754901
SOD	SOD-Cu-Zn	5	3	3	7	2	2	22	0.798952
**SOD**	**SOD-Mn-Fe**	**30**	**5**	**4**	**2**	**4**	**6**	**51**	**0.000052**
SPZ	SPZ	0	0	1	0	0	0	1	0.575875
**SRPN**	**SRPN-INHIB**	**26**	**8**	**8**	**2**	**8**	**4**	**56**	**0.001248**
SRPN	SRPN-nonINHIB	4	2	2	0	3	2	13	0.281764
TEP	TEP	1	1	2	1	1	4	10	0.376665
TOLLPATH	TUBE	0	0	0	0	0	1	1	0.562898
	**Totals**	**965**	**275**	**369**	**247**	**176**	**263**	**2295**	

Peptide sequences were harvested from ImmunoDB [24] for *An. gambiae*, *Ae. aegypti*, and *D. melanogaster *for each of the following gene families: All 8,020 EST clusters were blasted against a database of these peptides using blastx (e-value cutoff of 1e^-5^). BLAST hits were parsed from output files using tcl_blast_parser_123_V017.tcl [55] with a cut-off of 40% match, 1e^-20 ^normalized e-value, and a minimum match length of 30 residues. Top hits were taken for each significant match and verified manually. Matches then were categorized by family and subfamily according to Waterhouse et al. (2007) (Left column) and the composition (# of compiled sequences) of EST clusters were collated from the assembly according to the library(s) from which ESTs came: asuhem (bacteria-inoculated, hemocyte), diroinf (whole body, *Dirofilaria immitis *injected), imacbac (bacteria-inoculated whole body), brumal (blood-fed *Brugia malayi*), n7 and n14 (naïve, 5–7 and 12–14 days of age). Stekel R values [23] were determined for the groupings using IDEG6 [20] and those having a score of 0.001 or better were considered significantly differential, and are flagged with bolded and underlined text for the row.

This analysis underscores the degree to which immunity related ESTs are enriched in libraries from bacteria-inoculated mosquitoes. Particularly from the hemocyte library, ESTs from all subcategories are represented in abundance (Table 4). We expected to see some evidence of increased abundance of ESTs related to melanization, because published reports on the melanization response indicate that phenoloxidase is up-regulated as a result of immune-response activation [5]. However, few ESTs representing the biochemical pathway of melanogenesis were evident amongst the clusters (see Table 3). This limited representation could be a result of cloning bias inherent in library production, or introduced due to inoculation methodology, or even wound healing. Or, up-regulation may not be necessary to affect the response that we know to be occurring in the mosquito at the time points chosen for library construction [19].

### Comparisons with *Ae. aegypti*, *An. gambiae*, and *D. melanogaster*

The family Culicidae contains approximately 2,500 species of mosquitoes, of which only a handful are capable of vectoring disease. Much of the current effort to understand the molecular components of vector competence has focused on *An. gambiae *and *Ae. aegypti *[25,26], because these species transmit disease agents that have a tremendous impact on global public health (malaria, and dengue fever and yellow fever viruses, respectively). Comparative genomics analysis between these mosquitoes and the ongoing genome project on *Culex pipiens quinquefasciatus*, as compared to the fruit fly, have provided and will provide resources to bolster studies to systematically investigate common and mosquito species-specific gene function [25-28]. This includes gaining new insight into the molecular basis of insecticide resistance, host-seeking behaviour, blood feeding, and vector-parasite interactions that are unique to blood-feeding (hematophagous) vectors. The last of these is perhaps the most dramatic separation between the mosquitoes and fruit flies – hematophagy is intimately tied to a variety of physiologies including oogenesis and immunity, and therefore imposes unique demands on mosquitoes as compared to *Drosophila*. In a microarray analysis of *An. gambiae*, 25% of the genes on the array changed transcript levels in response to blood-feeding [29].

Among the Diptera, there is an evolutionary divergence of approximately 250 million years separating mosquitoes from *D. melanogaster*. The mosquitoes *An. gambiae *and *Ae. aegypti *are separated by 150 million years [26]. *An. gambiae *is a member of the subfamily Anophelinae, which contains the primary vectors of human malaria. In contrast, *Ae. aegypti *is a member of the subfamily Culicinae, which contains the majority of mosquito species that are of medical or veterinary importance, e.g., *Aedes, Culex, Armigeres*, and *Mansonia*. These two mosquito subfamilies differ significantly in genomic structure [30-32], and in vector competence. Broadly, *Anopheles *species are most often incriminated as vectors of parasitic disease agents (e.g., malaria and filarial worm parasites), and *Aedes *and *Culex *species are critically important in the transmission of arthropod-borne viruses as well as filarial worms.

*Ar. subalbatus *is a competent vector of viruses and parasites, and is more closely related to *Ae. aegypti *than to *An gambiae*; *Ae. aegypti *and *Ar. subalbatus *are phylogenetically linked at the level of tribe (Culicini). Therefore, comparisons between these two species of mosquito may provide unique insights into vector competence and innate immunity.

Based on the evolutionary distance, vector status, and vector competence of the fly species for which we have genome data, we asked: of the 8,020 EST clusters or singletons, how many have homologs in the available databases for 4 fly genomes/transcriptomes? The output from blastx analysis of predicted peptide sequences was filtered to search for homologous sequences using an e-value cutoff of 1e-20, a percent match of 40% (true matches, not conserved), and a minimum match length of 30 for the high-scoring segment pair. A large number of clusters (3,013 (38%)) did not have a homolog in any database as defined by this screen.

**Figure 3:**
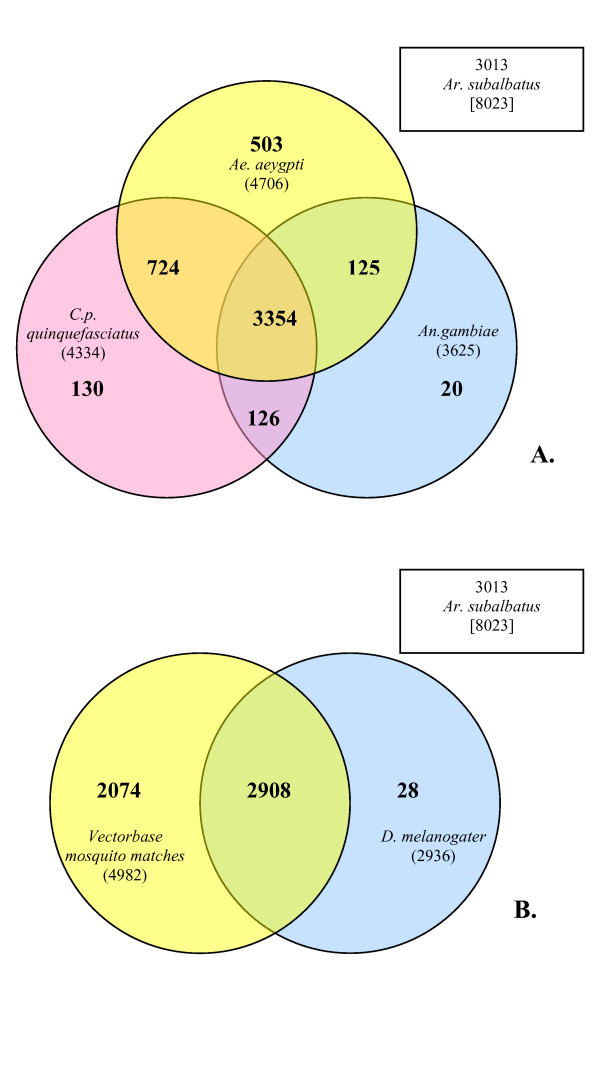
**Homologous sequences for *Ar. subalbatus *found in fly databases**. A) Comparative analysis of *Ar. subalbatu*s EST clusters with predicted peptides from 3 other mosquito species with completed genomes: *Ae. aegypt*i, *An. gambiae*, and *C.p. quinquefasciatus*. Overlapping regions indicate homologous sequences from blastx searches against the peptide databases. A homolog is defined as having an e-value cutoff of 1e-20, a percent match of 40% (true matches, not conserved), and a minimum match length of 30 for the high-scoring segment pair (HSP). This comparison includes 8,020 possible cluster sequences from *Ar. subalbatus *(brackets), of which 3,013 had no homolog. Boxes directly adjacent to circles indicate 1) the species being compared to *Ar. subalbatus*, and 2) the total # of homologous sequences between that species and *Ar. subalbatus*. B) A gene list of the total of overlapping and non-overlapping *Ar. subalbatus *homologs to *Ae. aegypt*i, *An. gambiae*, and *C. p. quinquefasciatus *was compared to a gene list of homologs found to *D. melanogaster*. A significant number of genes (2,074) from *Ar. subalbatus *have no homolog in the fruit fly, but qualify as homologs to genes in other mosquito species.

Those clusters that were homologous were subjected to Venn analysis (Figure 3A) to discover overlapping predicted peptides in 3 other mosquito species: *Ae. aegypt*i, (Ae Vectorbase AaegL1.1), *An. gambiae *(Anoph Vectorbase AgamP3), and *C.p. quinquefasciatus *(Cpip Vectorbase CpipJ1.0_5), and the fruit fly, *D. melanogaster*. The mosquito with the largest number of gene products that are uniquely homologous to *Ar. subalbatus *is *Ae. aegypti*, as would be predicted by the degree of relatedness of these two mosquitoes. In comparing *Ar. subalbatus *to all available mosquito and *Drosophila *homologous predicted peptides, 2908 sequences are represented in all fly species. A significant number (2,074) of clusters from *Ar. subalbatus *qualify as homologs to genes in other mosquito species, but have no homolog in the fruit fly (Figure 3B).

**Figure 4:**
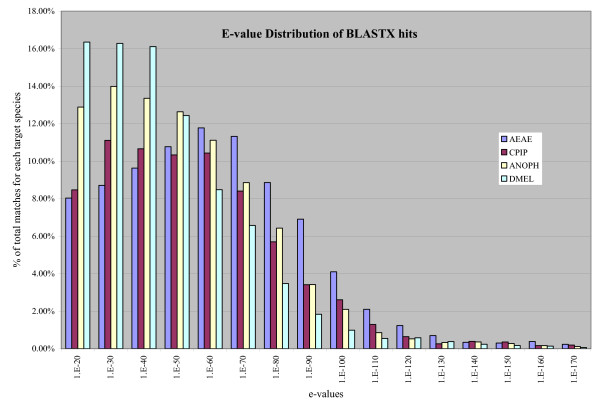
**Frequency distribution of the quality of blastx matches (according to e-value) for genes considered to be homologs in *Ae. aegypt*i (AEAE), *An. gambiae *(ANOPH), *C.p. quinquefasciatus *(CPIP), and *D. melanogaster *(DMEL)**. The number of e-values within a range is presented as a percentage of the total number of homologs per species. The graph shows an increasing trend of higher quality matches in more closely related species, while a majority of matches in distant species are lower quality.

Taking this one step further, from quantity of hits to quality of hits, we looked at the frequency distribution of e-value hits for the homologous sequences (Figure 4). There is an obvious shift toward more significant e-values for homologs in *Ae. aegypti*, a shift away from more significant e-values for homologs in *Drosophila *and *Anopheles*, and homologs in *Cx. pipiens *display an intermediate shift, closer to that seen in *Ae. aegypti*.

## Conclusion

Following recognition of any pathogen in a mosquito, a cascade of innate immune responses ensues that can include humoral responses (e.g. production of antimicrobial peptides), cellular (e.g. phagocytosis) and cell-mediated events (e.g. melanotic encapsulation). Because immunity-related genes function in concert to clear a pathogen [33,34], it is informative to use a holistic approach when evaluating expression and/or regulation i.e. it is likely that most of these genes are not activated independent of other immune-response genes. For example, in *Ar. subalbatus*, the biochemical pathway required for melanin biosynthesis is well characterized, but there is much to learn about the anti-filarial worm response as a whole in this mosquito species. What is readily apparent from the limited number of functional genomics studies that have investigated insect immunity, is that we really do not know very much about the mechanisms required to successfully eliminate an invading pathogen from a refractory mosquito (Aliota *et al*. [19]), and the subsequent changes necessary for a successful return to homeostasis.

There are a large number of unknown genes found in this and many other EST and microarray projects. We hypothesize that a large proportion of these unknowns are functionally linked to the unique and specific immune response of *Ar. subalbatus*, because of the material used to construct the libraries from which ESTs were produced. The rapidly expanding bank of large EST datasets and whole genome sequences for mosquitoes [1,26,28,35-39] provide the capability to critically evaluate the unknowns in the context of the many characterized facets of innate immunity, simultaneously. A microarray platform based on this *Ar. subalbatus *EST dataset has been designed for this purpose, and was screened with material from immune-response activated mosquitoes (Aliota *et al*. [19]).

For comparative purposes at the species level, this large dataset provides an important addition to the available sequence databases. Dipterans exhibit extraordinary variation in morphology, behaviour and physiology, so these ESTs add to the ongoing and increasingly powerful comparisons of fly species [29,40-42]. By virtue of hematophagy, mosquitoes are presented with unique physiologic challenges as compared to fruit files; at a minimum, blood-feeding requires host-seeking, triggers oogenesis, and exposes mosquitoes to a variety of blood-borne pathogens. Some of these challenges are shared with other vectors of disease agents. Vector-borne diseases such as malaria, leishmaniasis, African and American trypanosomiasis, Lyme disease and epidemic typhus, are caused by disease agents that are transmitted by mosquitoes, sandflies, tsetse, kissing bugs, ticks and body lice, respectively. There is a great deal of promise for enhancing our understanding of vector biology through genome sequencing and functional genomics analysis that will be increasingly available for a number of these species [43].

## Methods

### Mosquito maintenance

*Ar. subalbatus *was obtained from the University of Notre Dame in 1986. Larvae were hatched in distilled water and fed a ground slurry of Tetramin^® ^fish food. Pupae were separated by sex, and females transferred in lots of 80 to cartons. Adult females were fed on 0.3 M sucrose-soaked cotton balls. All mosquitoes were maintained at 26.5° ± 1°C, 75% ± 10% relative humidity with a 16 hr/8 hr light/dark cycle beginning and ending with a 90 min crepuscular period [1].

### Immune response activation and tissue collection

To construct libraries from immune response activated mosquitoes, 2–3 day old adult female *Ar. subalbatus *either were inoculated or infected with the pathogen or parasite known to elicit the response of interest.

*Bacteria inoculation*. A mixture of *E. coli *K12 and *M. luteus *was used as an inoculum as previously described [44]. Cold-immobilized mosquitoes were held in place with a vacuum saddle, and a 0.15 mm stainless steel probe was dipped into a bacterial pellet and inserted into the cervical membrane. Mosquitoes were returned to the insectary for 24, 48, or 72 h prior to harvesting.

*Dirofilaria immitis inoculation*. Cold-immobilized mosquitoes were secured in a vacuum saddle as previously described [45] and injected in the cervical membrane with approximately 20 *D. immitis *microfilarae (mf) in physiologic saline [46], and returned to the insectary for 24 or 48 hours prior to harvesting.

*Brugia malayi infection*. Sucrose was removed from the cartons 14–16 h prior to presenting mosquitoes to gerbils infected with *B. malayi *(microfilaraemia of approximately 44 mf/20 ml) for a blood meal. Gerbils were anesthetized with a ketamine/xylazine mixture. Microfilaremia was measured using 20 ul of blood collected by retro-orbital bleeding; formalin was added to lyse red blood cells and microfilariae were counted using phase microscopy as done previously [47]. Replete females were returned to the insectary for 24, 48, or 72 hours prior to harvesting.

*Naïve blood fed mosquitoes*: Sucrose was removed from the cartons 14–16 h prior to presenting mosquitoes to uninfected gerbils for a blood meal. Gerbils were anesthetized as described previously. Replete females were returned to the insectary for 24, 48, or 72 hours prior to harvesting. The library developed from this source did not produce quality sequences, so sequence data are unavailable.

*Naïve mosquitoes*. Females were randomly collected by aspiration from cartons of undisturbed, non-infected naïve adult females at 5–7 and 14–21 days post-eclosion.

### Tissue collection

RNA was isolated from 5–10 whole bodies for the following libraries: *E. coli *and *M. luteus *inoculated, *D. immitis *inoculated, *B. malayi *infected, naïve 7-day, naïve 14-day, and naïve bloodfed. For whole body collection, infected or inoculated female mosquitoes were collected at the aforementioned time points, frozen on dry ice, and stored at -80°C until ready for extraction. Frozen bodies were homogenized in a 1.5 ml tube using a Kontes^® ^tissue grinder in the presence of guanidinium thiocyanate-phenol-chloroform solution [48]. For hemocyte-derived bacteria inoculated libraries, a volume displacement method was used, as previously described [1]. One drop of perfusate was collected from each mosquito, kept on dry ice, and stored at -80°C until ready for extraction.

### Library construction

RNA was extracted from mosquito whole bodies or hemocytes by single-step guanidinium thiocyanate-phenol-chloroform extraction [48]. RNA was visualized on ethidium bromide-stained agarose gels to confirm quality, and then material from all time points were pooled. Complimentary DNA libraries were constructed using the SMART cDNA Library Construction kit (Clontech, Palo Alto, CA). Purified RNA was poly(A) selected for the long range PCR templates for whole body libraries, while total RNA was used for the hemocyte libraries.

### Sequence collection

For all libraries, sequence data were collected as previously described [1]. Briefly, plaques were blue/white screened, isolated by robotic picker, and used directly as a template in PCR reactions at the University of Wisconsin. At Yang-Ming the library was subjected to a mass excision protocol to produce plasmid templates for sequencing, as described in the manufacturer's protocol. The number of ESTs produced from either method is described in Table 1.

### EST clustering

A total of 44,940 trace files from both the UW and Yang-Ming collections were base-called and vector-trimmed using *phred *version 0.020425.c [49,50]. A "trim_cutoff" value of 0.025 was used to remove poor quality bases from the ends of reads, and SCF3 trace files were output for downstream clustering. Verified duplicate files from replicate sequencing were removed from the pool to reduce perceived cluster depth and improve data analysis. Poly-A tails and any remaining vector sequences were then removed with TIGR's *seqclean *[51], traces identified as contaminants from *E. coli *or any of the pathogens used for stimulus were removed, and finally, all traces with less than 51 bases of quality sequence were discarded, resulting in 38,079 traces proceeding into the assembler.

Quality trace data were clustered using LaserGene Seqman, Genome Edition (DNASTAR, Inc.) [52] on a WindowsXP workstation. A rapid, high stringency clustering was performed first, using the "Fast Assembler" module, with the following parameters: minimum match 90%, match size 25, match spacing 150, gap penalty 0, gap length penalty 0.7, end position mismatch 0, and minimum sequence length 50. These parameters are very conservative within the context of this program (i.e. minimizes false joins), so further automated merging with the "Classic Assembler" module was performed at a match size of 12, and a minimum match percentage of 90%. All other parameters were set to default. This had the effect of merging clusters that were very closely related with minimal gap sizes.

### Similarity searches

To predict gene products and assign gene ontology classifications, EST clusters were compared to sequences from the GenPept database (Genbank version 156) and gene products from the whole genome annotations of *D. melanogaster *(Flybase version 5.1), *An. gambiae *(ENSEMBL genebuild 41), and *Ae. aeygpti *(Vectorbase version L1.1). A FASTA-formatted file was collected from the assembly software, and subjected to BLASTX searches using the aforementioned databases. An E-value cut-off of 10^-3 ^was used to reduce non-informative hits, and filtering was not used. Search results were uploaded to A Systematic Annotation Package for Community Analysis of Genomes (ASAP) annotation workbench for manual annotation [53].

### Sequence annotation

The annotations of EST clusters in ASAP were conducted in a similar fashion as outlined previously [1], excluding protein domain searches due to the large data set size. Homolog information was collected for both *An. gambiae *(Ensembl) and *Ae. aeygpti *(Vectorbase), with links provided to those databases. Special attention was paid to Gene Ontology descriptors on the matches to *D. melanogaster *in Flybase. Where an annotation to Flybase was of "putative" or better, Gene Ontology information was transferred onto the cluster annotation.

### Data sharing

All data for this project are publicly accessible in ASAP via the web as annotated collapsed EST clusters [54]. Individual ESTs have been deposited with the National Center for Biotechnology Information (NCBI) dbEST: database of Expressed Sequence Tags, under the following accession number range: EU204979 – EU212998.

## Authors' contributions

GFM and LCB annotated sequences, analyzed data and prepared the text and figures. HYK analyzed sequence data. TAR constructed libraries and annotated sequence data. LCB and JFF prepared materials for library construction and annotated sequence data. GFM developed sequencing techniques and supervised sequencing efforts at UW-Madison. MTA contributed to manuscript preparation. IYT and CYH excised libraries for plasmid sequencing. TTL, KJH, SFT, and UCY conceived of and optimized parameters for plasmid sequencing. NTP facilitated the use of ASAP to annotate sequence data. WLC, BMC, and CCC conceived these studies and supervised all aspects of data collection and analysis.

## Supplementary Material

Additional file 1 Cluster analysis using six different statistical methods to determine differential copy numbers of ESTs broken down by library. Clusters and their constituent ESTs were analysed using IDEG6 [20] to find clusters where the number of ESTs were statistically different between the libraries. Each row represents the Genbank Accession number for cluster, which is linked to the corresponding record at NCBI for ease of access, and the columns are the number of ESTs from each of the six libraries that are a member of it. The libraries are: asuhem (bacteria-inoculated, hemocyte), diroinf (whole body, *Dirofilaria immitis *injected), imacbac (bacteria-inoculated whole body), brumal (blood-fed *Brugia malayi*), n7 and n14 (naïve, 5–7 and 12–14 days of age). Columns with (norm) in the header are the number of ESTs in that library normalized by the number of ESTs total in that library and the number of ESTs total. The "bluer" the shading, the more "up" the relative abundance of ESTs are compared the the rest of the libraries in that row. "AC" columns are Audic and Claverie 2 × 2 comparisons [21]; "Fisher" columns are Fishers Exact Test 2 × 2 comparisons; "Chi2 × 2" columns are Chi Square 2 × 2 comparison, and "GT" is Geller and Tobin scores [22]. The R value is the inverse log of the Stekel R Score [23], and the Chi value is a general Chi square analysis. Yellow shaded cells are filtered in the 95% or higher significance range. The other tab, "Flagged Differential" contains the same data as in Table 4, but include the "AC", "Fishers", "Chi2 × 2", and "GT" columns, and the Genbank accession numbers are linked to NCBI. The yellow cell shading in the product column indicates clusters that are considered differential in *Brugia malayi *blood-fed females in Aliota, et. al. [19] Click here for file

## References

[B1] Bartholomay LC, Cho WL, Rocheleau TA, Boyle JP, Beck ET, Fuchs JF, Liss P, Rusch M, Butler KM, Wu RC, Lin SP, Kuo HY, Tsao IY, Huang CY, Liu TT, Hsiao KJ, Tsai SF, Yang UC, Nappi AJ, Perna NT, Chen CC, Christensen BM (2004). Description of the transcriptomes of immune response-activated hemocytes from the mosquito vectors *Aedes aegypti* and Armigeres subalbatus. Infect Immun.

[B2] Dimopoulos G, Richman A, Muller HM, Kafatos FC (1997). Molecular immune responses of the mosquito Anopheles gambiae to bacteria and malaria parasites. Proc Natl Acad Sci U S A.

[B3] Vernick KD, Oduol F, Lazzaro BP, Glazebrook J, Xu J, Riehle M, Li J (2005). Molecular genetics of mosquito resistance to malaria parasites. Curr Top Microbiol Immunol.

[B4] Dimopoulos G, Muller HM, Levashina EA, Kafatos FC (2001). Innate immune defense against malaria infection in the mosquito. Curr Opin Immunol.

[B5] Christensen BM, Li J, Chen CC, Nappi AJ (2005). Melanization immune responses in mosquito vectors. Trends Parasitol.

[B6] Garnham PCC (1966). Malaria parasites and other haemosporidia.

[B7] Chen WJ, Dong CF, Chiou LY, Chuang WL (2000). Potential role of Armigeres subalbatus (Diptera: Culicidae) in the transmission of Japanese encephalitis virus in the absence of rice culture on Liu-chiu islet, Taiwan. J Med Entomol.

[B8] Kanojia PC, Geevarghese G (2005). New mosquito records of an area known for Japanese encephalitis hyperendemicity, Gorakhpur District, Uttar Pradesh, India. J Am Mosq Control Assoc.

[B9] Hillyer JF, Schmidt SL, Christensen BM (2004). The antibacterial innate immune response by the mosquito *Aedes aegypti* is mediated by hemocytes and independent of Gram type and pathogenicity. Microbes Infect.

[B10] Hillyer JF, Schmidt SL, Christensen BM (2003). Hemocyte-mediated phagocytosis and melanization in the mosquito Armigeres subalbatus following immune challenge by bacteria. Cell Tissue Res.

[B11] Hillyer JF, Schmidt SL, Christensen BM (2003). Rapid phagocytosis and melanization of bacteria and Plasmodium sporozoites by hemocytes of the mosquito *Aedes aegypti*. The Journal of parasitology.

[B12] Hernandez S, Lanz H, Rodriguez MH, Torres JA, Martinez-Palomo A, Tsutsumi V (1999). Morphological and cytochemical characterization of female Anopheles albimanus (Diptera: Culicidae) hemocytes.. J Med Entomol.

[B13] Hernandez-Martinez S, Lanz H, Rodriguez MH, Gonzalex-Ceron L, Tsutsumi V (2002). Cellular-mediated reactions to foreign organisms inoculated into the hemocoel of Anopheles albimanus (Diptera: Culicidae). J Med Entomol.

[B14] Irving P, Troxler L, Heuer TS, Belvin M, Kopczynski C, Reichhart JM, Hoffmann JA, Hetru C (2001). A genome-wide analysis of immune responses in Drosophila. Proc Natl Acad Sci U S A.

[B15] Lowenberger C (2001). Innate immune response of *Aedes aegypti*. Insect Biochem Mol Biol.

[B16] Infanger LC, Rocheleau TA, Bartholomay LC, Johnson JK, Fuchs J, Higgs S, Chen CC, Christensen BM (2004). The role of phenylalanine hydroxylase in melanotic encapsulation of filarial worms in two species of mosquitoes. Insect Biochem Mol Biol.

[B17] Ferdig MT, Beerntsen BT, Spray FJ, Li J, Christensen BM (1993). Reproductive costs associated with resistance in a mosquito-filarial worm system. Am J Trop Med Hyg.

[B18] Wang X, Rocheleau TA, Fuchs JF, Hillyer JF, Chen CC, Christensen BM (2004). A novel lectin with a fibrinogen-like domain is involved in the innate immune response of Armigeres subalbatus against bacteria. Insect Mol Biol.

[B19] Aliota MT, Fuchs JF, Mayhew GF, Chen CC, Christensen BM (2007). Mosquito transcriptome changes and filarial worm resistance in Armigeres subalbatus. BMC Genomics.

[B20] Romualdi C, Bortoluzzi S, D'Alessi F, Danieli GA (2003). IDEG6: a web tool for detection of differentially expressed genes in multiple tag sampling experiments. Physiological genomics.

[B21] Audic S, Claverie JM (1997). The significance of digital gene expression profiles. Genome research.

[B22] Greller LD, Tobin FL (1999). Detecting selective expression of genes and proteins. Genome research.

[B23] Stekel DJ, Git Y, Falciani F (2000). The comparison of gene expression from multiple cDNA libraries. Genome research.

[B24] Insect Immune-Related Genes and Gene Families. http://cegg.unige.ch/Insecta/immunodb.

[B25] Holt RA, SL. H, Subramanian GM, Halpern A, Sutton GG, Charlab R, Nusskern DR, Wincker P, Clark AG, Ribeiro JM, Wides R, Salzberg SL, Loftus B, Yandell M, Majoros WH, Rusch DB, Lai Z, Kraft CL, Abril JF, Anthouard V, Arensburger P, Atkinson PW, Baden H, de Berardinis V, Baldwin D, Benes V, Biedler J, Blass C, Bolanos R, Boscus D, Barnstead M, Cai S, Center A, Chaturverdi K, Christophides GK, Chrystal MA, Clamp M, Cravchik A, Curwen V, Dana A, Delcher A, Dew I, Evans CA, Flanigan M, Grundschober-Freimoser A, Friedli L, Gu Z, Guan P, Guigo R, Hillenmeyer ME, Hladun SL, Hogan JR, Hong YS, Hoover J, Jaillon O, Ke Z, Kodira C, Kokoza E, Koutsos A, Letunic I, Levitsky A, Liang Y, Lin JJ, Lobo NF, Lopez JR, Malek JA, McIntosh TC, Meister S, Miller J, Mobarry C, Mongin E, Murphy SD, O'Brochta DA, Pfannkoch C, Qi R, Regier MA, Remington K, Shao H, Sharakhova MV, Sitter CD, Shetty J, Smith TJ, Strong R, Sun J, Thomasova D, Ton LQ, Topalis P, Tu Z, Unger MF, Walenz B, Wang A, Wang J, Wang M, Wang X, Woodford KJ, Wortman JR, Wu M, Yao A, Zdobnov EM, Zhang H, Zhao Q, Zhao S, Zhu SC, Zhimulev I, Coluzzi M, della Torre A, Roth CW, Louis C, Kalush F, Mural RJ, Myers EW, Adams MD, Smith HO, Broder S, Gardner MJ, Fraser CM, Birney E, Bork P, Brey PT, Venter JC, Weissenbach J, Kafatos FC, Collins FH (2002). The genome sequence of the malaria mosquito Anopheles gambiae. Science.

[B26] Nene V, Wortman JR, Lawson D, Haas B, Kodira C, Tu ZJ, Loftus B, Xi Z, Megy K, Grabherr M, Ren Q, Zdobnov EM, Lobo NF, Campbell KS, Brown SE, Bonaldo MF, Zhu J, Sinkins SP, Hogenkamp DG, Amedo P, Arsenburger P, Atkinson PW, Bidwell S, Biedler J, Birney E, Bruggner RV, Costas J, Coy MR, Crabtree J, Crawford M, Debruyn B, Decaprio D, Eiglmeier K, Eisenstadt E, El-Dorry H, Gelbart WM, Gomes SL, Hammond M, Hannick LI, Hogan JR, Holmes MH, Jaffe D, Johnston SJ, Kennedy RC, Koo H, Kravitz S, Kriventseva EV, Kulp D, Labutti K, Lee E, Li S, Lovin DD, Mao C, Mauceli E, Menck CF, Miller JR, Montgomery P, Mori A, Nascimento AL, Naveira HF, Nusbaum C, O'Leary S B, Orvis J, Pertea M, Quesneville H, Reidenbach KR, Rogers YH, Roth CW, Schneider JR, Schatz M, Shumway M, Stanke M, Stinson EO, Tubio JM, Vanzee JP, Verjovski-Almeida S, Werner D, White O, Wyder S, Zeng Q, Zhao Q, Zhao Y, Hill CA, Raikhel AS, Soares MB, Knudson DL, Lee NH, Galagan J, Salzberg SL, Paulsen IT, Dimopoulos G, Collins FH, Bruce B, Fraser-Liggett CM, Severson DW (2007). Genome sequence of *Aedes aegypti*, a major arbovirus vector. Science.

[B27] Mongin E, Louis C, Holt RA, Birney E, Collins FH (2004). The Anopheles gambiae genome: an update. Trends Parasitol.

[B28] Waterhouse RM, Kriventseva EV, Meister S, Xi ZY, Alvarez KS, Bartholomay LC, Barillas-Mury C, Bian G, Blandin S, Christensen BM, Dong Y, Jiang H, Kanost M, Koutsos AC, Levashina EA, Li J, Ligoxygakis P, MacCallum MR, Mayhew GF, Mendes A, Michel K, Osta MA, Paskewitz S, Shin SW, Vlachou D, Wang L, Wei W, Zheng L, Zou Z, Severson DW, Raikhel A, Kafatos FC, Dimopoulos G, Zdobnov EM, Christophides GK (2007). Evolutionary dynamics of immune-related genes and pathways in disease-vector mosquitoes. Science.

[B29] Dana AN, Hong YS, Kern MK, Hillenmeyer ME, Harker BW, Lobo NF, Hogan JR, Romans P, Collins FH (2005). Gene expression patterns associated with blood-feeding in the malaria mosquito Anopheles gambiae. BMC Genomics.

[B30] Kaufman TC, Severson DW, Robinson GE (2002). The Anopheles genome and comparative insect genomics. Science.

[B31] Chambers EW, Lovin DD, Severson DW (2003). Utility of comparative anchor-tagged sequences as physical anchors for comparative genome analysis among the Culicidae. Am J Trop Med Hyg.

[B32] Severson DW, DeBruyn B, Lovin DD, Brown SE, Knudson DL, Morlais I (2004). Comparative genome analysis of the yellow fever mosquito *Aedes aegypti* with Drosophila melanogaster and the malaria vector mosquito Anopheles gambiae. J Hered.

[B33] Bartholomay LC, Mayhew GF, Fuchs JF, Rocheleau TA, Erickson SM, Aliota MT, Christensen BM (2007). Profiling infection responses in the hemocytes of the mosquito, *Aedes aegypti*.. Insect Mol Biol.

[B34] Hillyer JF, Christensen BM (2005). Mosquito phenoloxidase and defensin localization. J Histochem Cytochem.

[B35] Dimopoulos G, Casavant TL, Chang S, Scheetz T, Roberts C, Donohue M, Schultz J, Benes V, Bork P, Ansorge W, Soares MB, Kafatos FC (2000). Anopheles gambiae pilot gene discovery project: Identification of mosquito innate immunity genes from expressed sequence tags generated from immune-competent cell lines. Proc Natl Acad Sci U S A.

[B36] Sanders HR, Evans AM, Ross LS, Gill SS (2003). Blood meal induces global changes in midgut gene expression in the disease vector, *Aedes aegypti*. Insect Biochem Mol Biol.

[B37] Valenzuela JG, Francischetti IMB, Pham VM, Garfield MK, Ribeiro JMC (2003). Exploring the salivary gland transcriptome and proteome of the Anopheles stephensi mosquito. Insect Biochem Mol Biol.

[B38] Valenzuela JG, Pham VM, Garfield MK, Francishetti IMB, Ribiero JMC (2002). Toward a description of the sialome of the adult female mosquito *Aedes aegypti*. Insect Biochem Mol Biol.

[B39] Christophides GK (2002). Immunity-related genes and gene families in Anopheles gambiae. Science.

[B40] Biron DG, Joly C, Marche L, Galeotti N, Calcagno V, Schmidt-Rhaesa A, Renault L, Thomas F (2005). First analysis of the proteome in two nematomorph species, Paragordius tricuspidatus (Chordodidae) and Spinochordodes tellinii (Spinochordodidae). Infection, Genetics and Evolution.

[B41] Catteruccia F (2007). Malaria vector control in the third millennium: progress and perspectives of molecular approaches. Pest Manag Sci.

[B42] Dong Y, Aguilar R, Xi Z, Warr E, Mongin E, Dimopoulos G (2006). Anopheles gambiae immune responses to human and rodent Plasmodium parasite species. PLoS Pathog.

[B43] Lawson D, Arensburger P, Atkinson P, Besansky NJ, Bruggner RV, Butler R, Campbell KS, Christophides GK, Christley S, Dialynas E, Emmert D, Hammond M, Hill CA, Kennedy RC, Lobo NF, MacCallum MR, Madey G, Megy K, Redmond S, Russo S, Severson DW, Stinson EO, Topalis P, Zdobnov EM, Birney E, Gelbart WM, Kafatos FC, Louis C, Collins FH (2007). VectorBase: a home for invertebrate vectors of human pathogens. Nucl Acids Res.

[B44] Lowenberger CA, Smartt CT, Bulet P, Ferdig MT, Severson DW, Hoffmann JA, Christensen BM (1999). Insect immunity: molecular cloning, expression, and characterization of cDNAs and genomic DNA encoding three isoforms of insect defensin in *Aedes aegypti*. Insect Mol Biol.

[B45] Beerntsen BT, Christensen BM (1990). Dirofilaria immitis: effect on hemolymph polypeptide synthesis in *Aedes aegypti* during melanotic encapsulation reactions against microfilariae. Exp Parasitol.

[B46] Hayes RO (1953). Determination of a physiological saline solution for *Aedes aegypti* (L.). J Econ Entomol.

[B47] Beerntsen BT, Lowenberger CA, Klinkhammer JA, Christensen LA, Christensen BM (1996). Influence of anesthetics on the peripheral blood microfilaremia of Brugia malayi in the Mongolian jird, Meriones unguiculatus. The Journal of parasitology.

[B48] Chomczynski P, Sacchi N (1987). Single-step method of RNA isolation by acid guanidinium thiocyanate-phenol-chloroform extraction. Anal Biochem.

[B49] Ewing B, Green P (1998). Base-calling of automated sequencer traces using phred. II. Error probabilities. Genome research.

[B50] Ewing B, Hillier L, Wendl MC, Green P (1998). Base-calling of automated sequencer traces using phred. I. Accuracy assessment. Genome research.

[B51] Pertea G, Huang X, Liang F, Antonescu V, Sultana R, Karamycheva S, Lee Y, White J, Cheung F, Parvizi B, Tsai J, Quackenbush J (2003). TIGR Gene Indices clustering tools (TGICL): a software system for fast clustering of large EST datasets. Bioinformatics.

[B52] Burland TG (2000). DNASTAR's Lasergene sequence analysis software. Methods Mol Biol.

[B53] Glasner JD, Liss P, Plunkett III G, Darling A, Prasad T, Rusch M, Byrnes A, Gilson M, Beihl B, Blattner FR, Perna NT (2003). ASAP, a systematic annotation package for community analysis of genomes. Nucleic Acids Res.

[B54] A Systematic Annotation Package for Community Analysis of Genomes. https://asap.ahabs.wisc.edu/asap/logon.php.

[B55] BLAST Parser, Distance Matrix File And Protein Sequence Clustering. http://cgpdb.ucdavis.edu/BlastParser/Blast_Parser.html.

